# A Systematic Review and Meta-Analysis of the Relationship Between the Radiation Absorbed Dose to the Thyroid and Response in Patients Treated with Radioiodine for Graves' Disease

**DOI:** 10.1089/thy.2021.0302

**Published:** 2021-12-16

**Authors:** Jan Taprogge, Paul M.D. Gape, Lily Carnegie-Peake, Iain Murray, Jonathan I. Gear, Francesca Leek, Steve L. Hyer, Glenn D. Flux

**Affiliations:** ^1^ Joint Department of Physics, Royal Marsden NHSFT, Sutton, United Kingdom.; ^2^ The Institute of Cancer Research, London, United Kingdom.; ^3^ Department of Endocrinology, Epsom and St Helier University Hospitals NHS Trust, Carshalton, Surrey, United Kingdom.

**Keywords:** Graves', disease, meta-analysis, radiation absorbed dose, radioiodine, systematic review

## Abstract

***Background:*** Patients with Graves' disease are commonly treated with radioiodine. There remains controversy over whether the aim of treatment should be to achieve euthyroidism or hypothyroidism, and whether treatments should be administered with standard levels of radioactivity or personalized according to the radiation absorbed doses delivered to the thyroid. The aim of this review was to investigate whether a relationship exists between radiation absorbed dose and treatment outcome.

***Methods:*** A systematic review and meta-analysis of all reports published before February 13, 2020, were performed using PubMed, Web of Science, OVID MEDLINE, and Embase. Proportion of patients achieving nonhyperthyroid status was the primary outcome. Secondary outcomes were proportion of patients who were specifically euthyroid or hypothyroid. A random-effects meta-analysis of proportions was performed for primary and secondary outcomes, and the impact of the radiation absorbed dose on treatment outcome was assessed through meta-regression. The study is registered with PROSPERO (CRD42020175010).

***Results:*** A total of 1122 studies were identified of which 15, comprising 2303 Graves' disease patients, were eligible for the meta-analysis. A strong association was found between radiation absorbed dose and nonhyperthyroid and hypothyroid outcomes (odds ratio [OR] = 1.11 [95% confidence interval {CI} 1.08–1.14] and OR = 1.09 [CI 1.06–1.12] per 10 Gy increase). Higher rates of euthyroid outcome were found for radiation absorbed doses within the range 120–180 Gy when compared with outside this range (*n* = 1172, OR = 2.50 [CI 1.17–5.35], *p* = 0.018). A maximum euthyroid response of 38% was identified at a radiation absorbed dose of 128 Gy.

***Conclusions:*** The presented radiation absorbed dose–response relationships can facilitate personalized treatment planning for radioiodine treatment of patients with Graves' disease. Further studies are required to determine how patient-specific covariates can inform personalized treatments.

## Introduction

Hyperthyroidism has been widely treated with [^131^I]NaI (radioiodine) since 1941 (1). However, debate continues as to whether the aim of treatment should be to achieve hypothyroidism or euthyroidism (2–6). Additionally there is a lack of consensus on the optimal strategy to achieve either outcome. The most common approach is based on the administration of standard levels of radioactivity. However, a personalized approach based on calculated activities to deliver a specified radiation absorbed dose to the thyroid may deliver a euthyroid outcome where required (3). Recent guidelines from the National Institute for Health and Care Excellence highlighted the lack of randomized controlled trials (RCTs) in the use of radioiodine for the treatment of benign thyroid disease (6).

The aim of treatment of hyperthyroidism remains controversial. The American Thyroid Association (4) and the European Thyroid Association (5) recommend a single administration of radioactivity sufficient to render the patient hypothyroid (typically between 370 and 555 MBq). However, the European Association of Nuclear Medicine (EANM) guidelines (3,7) consider hypothyroidism a side effect of the treatment (8,9), which requires life-long thyroid hormone replacement and regular thyrotropin monitoring.

An audit of local general practitioners in the United Kingdom found that 21% of patients were over treated with the thyroid replacement drug levothyroxine, while undertreatment was observed in 9% of patients (10). Both outcomes potentially have negative health impacts for patients. A patient survey conducted by the British Thyroid Foundation found that ∼80% of patients were dissatisfied with their medication (11). The EANM guidelines state that treatment according to disease-specific prescribed radiation doses may achieve a euthyroid state, whereby the patient would not require thyroid hormone replacement (3).

Treatment protocols are currently based on evidence from single-center studies and vary widely. In performing this review, we aimed to consolidate the current literature regarding radiation absorbed doses to the thyroid for radioiodine treatment of hyperthyroidism and to investigate whether a relationship exists between these radiation absorbed doses and treatment outcome.

## Materials and Methods

### Search strategy and selection criteria

A comprehensive systematic review and meta-analysis of published studies were performed to evaluate the clinical outcomes of radioiodine therapy for hyperthyroidism with respect to the radiation absorbed doses to the thyroid. Articles published before February 13, 2020, were included. No restrictions were applied on language or type of study design. Only studies were included that reported radiation absorbed dose to the thyroid, follow-up time, and treatment outcomes for adult patients. Only full-text articles published in peer-reviewed journals were assessed.

PubMed, Web of Science, OVID MEDLINE, and Embase were searched following the principles and checklist provided by PRISMA (preferred reporting items for systematic reviews and meta-analyses) (12). The databases were searched for the following terms: (“iodine” OR “radioiodine” OR “I131” OR “I-131” OR “131I”) AND (“graves' disease” OR “hyperthyroidism”) AND (“dosimetry” OR “absorbed dose”). Study authors were not contacted and trial registries were not searched. Details of the protocol for this systematic review were registered on PROSPERO (CRD42020175010). Ethical approval was not relevant for this study, since it is solely based on literature.

Two reviewers (J.T. and G.D.F.) performed the initial search and screened results for duplicates. Two blinded reviewers (J.T. and G.D.F.) screened the remaining studies based on title and abstract for inclusion. Discrepancies between the selected studies were resolved as a joint decision by the two reviewers. Four reviewers (J.T., G.D.F., L.C.P., and P.M.D.G.) extracted data independently and collated the results in MS Excel spreadsheets.

Data were extracted on a subpopulation level for each treatment arm, corresponding to different radiation doses to the thyroid, where available. Data were extracted for the full study population in cases where data for different treatment arms were not reported.

### Data analysis

For each study, the following variables were extracted: number of subjects, disease type, discontinuation of antithyroid medication before treatment (yes-all/yes-some/none), presence of ophthalmopathy (yes-all/yes-some/none), follow-up period (months), median or mean age (years), proportion of male patients (percentage), median or mean amount of radioactivity (MBq), radiation absorbed dose to the thyroid (Gy), and proportion of patients euthyroid/hypothyroid/hyperthyroid at all follow-up times (percentage). The aim of treatment was recorded as either nonhyperthyroid (encompassing both euthyroid and hypothyroid), specifically euthyroid, or specifically hypothyroid. Dosimetry methodology was also extracted.

The main summary measures used were proportions of patients (with confidence intervals [95% CIs]) reaching specific endpoints after radioiodine treatment, relative to the size of the treatment arm subpopulation. The primary outcome used was proportion of patients who were nonhyperthyroid. Secondary outcomes were proportion of patients who were specifically euthyroid or hypothyroid. These were taken to be mutually exclusive and were individually defined in each study. Where the proportion of patients with euthyroid outcome was not reported, the proportion was determined as the difference between the patients rendered nonhyperthyroid and hypothyroid. Patients who required further radioiodine treatment were classed as hyperthyroid at follow-up.

Two reviewers (J.T. and L.C.P.) assessed risk of bias on a study level using the critical appraisal checklist developed by the Joanna Briggs Institute (13). Studies were classed as having a low, intermediate, or high risk of bias and studies were only included if classed as having low or intermediate risk of bias in the further data synthesis.

The meta-analysis was performed separately for Graves' disease and for any other hyperthyroid conditions. Only the response at last follow-up was included for the meta-analysis. The majority of included studies were uncontrolled and retrospective. Therefore, a random-effects meta-analysis of proportions was performed for nonhyperthyroid, euthyroid, and hypothyroid outcomes. DerSimonian and Laird's method was employed with a logit transformation (14,15). The I^2^ test was used to assess heterogeneity between studies. Meta-regression was performed to assess the impact of the extracted variables on the odds of achieving the respective outcomes. For the euthyroid outcome, where a nonmonotonic relationship is expected (16), a categorical variable was included to represent whether the radiation absorbed dose was within or outside a particular range.

Dose–response relationships were fitted based on a two-parameter log-logistic model (17) using the maximum likelihood principle for the nonhyperthyroid and hypothyroid outcomes. A sensitivity analysis was performed to identify whether results remained significant if only studies classed as having low risk of bias were included.

All statistical analyses were performed using R Statistical Software (version 3.5.2; R Foundation for Statistical Computing, Vienna, Austria) and the add-on package drc (18). The value *p* < 0.05 was considered statistically significant.

## Results

**FIG. 1.**
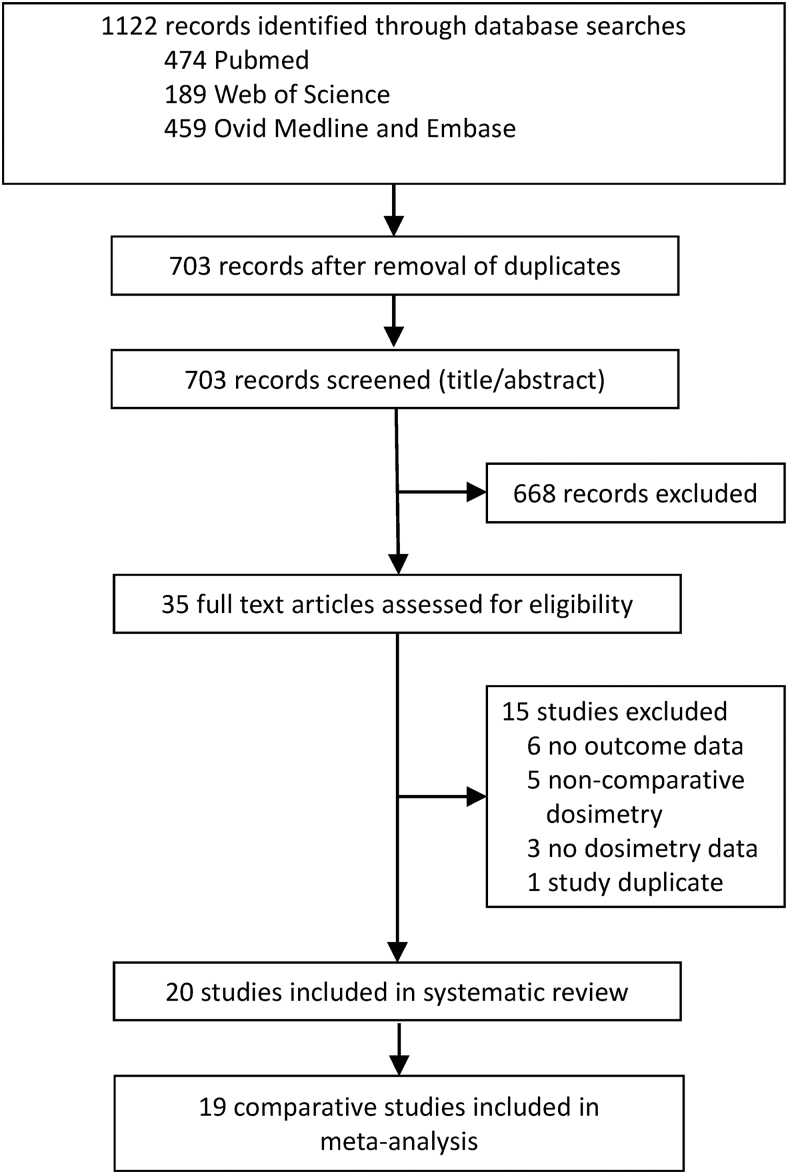
Flowchart for the systematic literature review.

A total of 1122 studies were identified for the systematic review of which 419 were excluded due to presentation of duplicate data. A further 668 studies were excluded for not satisfying the eligibility criteria based on title and abstract. Of the remaining 35 studies, a total of 20 full-text articles (16,19–37) were deemed eligible for the systematic review following independent analysis (Fig. 1). A summary of the study characteristics is presented in Table 1. Thirteen studies reported a patient cohort with Graves' disease, 5 reported a mixture of hyperthyroid conditions including Graves' disease, 1 study reported only hyperfunctioning thyroid nodules, and 1 study considered only patients with toxic nodular goiter.

**Table 1.** Characteristics of Included Studies

	# (disease types)	ATD	FU, months	Age, years	Male sex, %	OP	PrevRAI	Radiation absorbed dose, Gy	Rad Act Admin, MBq	Rates, %		
										HYPO	EU	HYPER
Amato *et al.* (2016, Italy) (19)	69 (GD, TA, TNG)	Yes	47	M 64 (SD 13)	36	NR	NR	M 223 (SD 49)	M 303 (SD 135)	23	72	4
Bajnok *et al.* (1999, Hungary) (20)	76 (GD)	Yes	6	M 49 (SD 12)^*^	18^*^	YS	YS	70	M 315 (SD 233)^*^	20	55	25
Bajnok *et al.* (1999, Hungary) (20)	29 (GD)	Yes	6	M 49 (SD 12)^*^	18^*^	YS	YS	M 90 (Rg 80–100)	M 315 (SD 233)^*^	34	38	28
Bajnok *et al.* (1999, Hungary) (20)	68 (GD)	Yes	12	M 49 (SD 12)^*^	18^*^	YS	YS	70	M 315 (SD 233)^*^	25	51	24
Bajnok *et al.* (1999, Hungary) (20)	25 (GD)	Yes	12	M 49 (SD 12)^*^	18^*^	YS	YS	M 90 (Rg 80–100)	M 315 (SD 233)^*^	44	40	16
Berg *et al.* (1996, Sweden) (21)	191 (GD)	Yes	5	Rg 29–70^*^	18	YS	NR	M 110 (Rg 100–120)	M 386 (SD 136)	NR	NR	7
Berg *et al.* (1996, Sweden) (21)	45 (TNG)	Yes	5	Rg 29–70^*^	4	YS	NR	M 110 (Rg 100–120)	M 461 (SD 115)	NR	NR	7
Blahd and Hays (1972, United States) (22)	241 (GD)	NR	NR	M 42 (Rg 21–78)	100	YS	YS	55	M 206 (SD 110)	NR	NR	45
Bockisch *et al.* (1993, Germany) (23)	14 (TA)	No	12	NR	NR	NR	NR	M 150 (Rg 120–180)	Rg 190–1100^*^	7	71	21
Bockisch *et al.* (1993, Germany) (23)	21 (TA, HTN, HN, GD, EuG)	No	12	NR	NR	NR	NR	M 100 (Rg 80–120)	Rg 190–1100^*^	5	76	19
Camps *et al.* (1996, Netherlands) (24)	39 (GD)	Yes	12	M 40 (Rg 11–80)	22	YS	NR	M 81 (Rg 24–163)	M 155 (Rg 54–940)	26	48	26
Camps *et al.* (1996, Netherlands) (24)	22 (TNG)	Yes	12	M 67 (Rg 24–90)	9	YS	NR	M 160 (Rg 38–317)	M 715 (Rg 78–1654)	9	59	32
Catargi *et al.* (1999, France) (25)	100 (GD)	NA	72	M 52 (SD 12)	11	NR	No	M 83 (Rg 36–232)	NR	41	26	33
Flower *et al.* (1994, UK) (26)	15 (GD)	NR	6	NR	NR	NR	No	M 10 (Rg 0–20)	M 75 (SD NR)	0	7	93
Flower *et al.* (1994, UK) (26)	27 (GD)	NR	6	NR	NR	NR	No	M 30 (Rg 20–40)	M 75 (SD NR)	0	26	74
Flower *et al.* (1994, UK) (26)	9 (GD)	NR	6	NR	NR	NR	No	M 50 (Rg 40–60)	M 75 (SD NR)	11	0	89
Flower *et al.* (1994, UK) (26)	14 (GD)	NR	6	NR	NR	NR	No	M 70 (Rg 60–80)	M 75 (SD NR)	14	14	71
Grosso *et al.* (2005, Italy) (27)	32 (GD)	Yes	12	M 61 (SD 13)	24^*^	YS	NR	M 148 (SD 26)	M 455 (SD 250)	25	59	16
Grosso *et al.* (2005, Italy) (27)	58 (GD)	Yes	12	M 54 (SD 14)	24^*^	YS	NR	M 295 (SD 52)	M 444 (SD 181)	40	47	14
Howarth *et al.* (2001, Australia) (28)	28 (GD)	Yes	6	M 46 [CI 42–52]	14^*^	YS	NR	60	M 154 [CI 119–190]	7	32	61
Howarth *et al.* (2001, Australia) (28)	29 (GD)	Yes	6	M 42 [CI 37–45]	14^*^	YS	NR	90	179 [CI 148–210]	17	24	59
Hyer *et al.* (2018, UK) (29)	284 (GD)	Yes	18	Md 46 (Rg 18–81)^*^	24^*^	YS	No	Md 56 [CI 55–58]	Md 81 (Rg 17–1377)	9	44	47
Hyer *et al.* (2018, UK) (29)	284 (GD)	Yes	36	Md 46 (Rg 18–82)^*^	24^*^	YS	No	Md 56 [CI 55–58]	Md 81 (Rg 17–1377)	13	41	46
Hyer *et al.* (2018, UK) (29)	284 (GD)	Yes	60	Md 46 (Rg 18–82)^*^	24^*^	YS	No	Md 55 [CI 55–58]	Md 81 (Rg 17–1377)	17	38	45
Hyer *et al.* (2018, UK) (29)	284 (GD)	Yes	120	Md 46 (Rg 18–82)^*^	24^*^	YS	No	Md 56 [CI 55–58]	Md 81 (Rg 17–1377)	21	30	49
Kobe *et al.* (2008, Germany) (30)	30 (GD)	Yes	12	Md 48 (Rg 18–80)^*^	17^*^	YS	NR	M 190 (SD NR)	NR	NR	NR	7
Kobe *et al.* (2008, Germany) (30)	137 (GD)	Yes	12	Md 48 (Rg 18–80)^*^	17^*^	YS	NR	M 231 (Rg 206–255)	NR	NR	NR	4
Kobe *et al.* (2008, Germany) (30)	181 (GD)	Yes	12	Md 48 (Rg 18–80)^*^	17^*^	YS	NR	M 281 (Rg 256–305)	NR	NR	NR	4
Kobe *et al.* (2008, Germany) (30)	128 (GD)	Yes	12	Md 48 (Rg 18–80)^*^	17^*^	YS	NR	M 331 (Rg 306–355)	NR	NR	NR	2
Kobe *et al.* (2008, Germany) (30)	50 (GD)	Yes	12	Md 48 (Rg 18–80)^*^	17^*^	YS	NR	M 381 (Rg 356–405)	NR	NR	NR	2
Orsini *et al.* (2012, Italy) (31)	29 (GD)	Yes	12	M 53 (SD 18)^*^	29^*^	No	No	100	NR	NR	NR	52
Orsini *et al.* (2012, Italy) (31)	25 (GD)	Yes	12	M 53 (SD 18)^*^	29^*^	No	No	200	NR	NR	NR	36
Orsini *et al.* (2012, Italy) (31)	29 (GD)	Yes	12	M 53 (SD 18)^*^	29^*^	No	No	M 407 (SD 23)	M 524 (SD 201)	93	3	3
Oszukowska *et al.* (2010, Poland) (32)	40 (GD, TNG)	Yes	6	M 52 (SD 13)^*^	15^*^	No	NR	M 175 (Rg 150–200)	NR	20	35	45
Oszukowska *et al.* (2010, Poland) (32)	40 (GD, TNG)	NA	6	M 52 (SD 13)^*^	15^*^	No	NR	M 175 (Rg 150–200)	NR	18	60	23
Oszukowska *et al.* (2010, Poland) (32)	40 (GD)	NA	6	M 52 (SD 13)^*^	15^*^	Yes	NR	M 300 (Rg 250–350)	NR	58	30	13
Oszukowska *et al.* (2010, Poland) (32)	40 (GD)	NA	6	M 52 (SD 13)^*^	15^*^	No	NR	M 300 (Rg 250–350)	NR	43	28	30
Peters *et al.* (1995, Germany) (16)	107 (GD)	Yes	6	Md 52 (Rg 31–80)	13	YS	YS	Md 119 Gy (Q25 = 90 Gy, Q75 = 154 Gy)	Md 298 (Q25 = 184, Q75 = 555)	23	35	42
Reinhardt *et al.* (2002, Germany) (33)	84 (GD)	YS	16	M 60 (SD 14)	29^*^	YS	NR	M 177 (SD 49)	M 570 (SD 285)	27	45	27
Reinhardt *et al.* (2002, Germany) (33)	78 (GD)	YS	15	M 58 (SD 15)	29^*^	YS	NR	M 236 (SD 53)	M 680 (SD 310)	33	44	23
Reinhardt *et al.* (2002, Germany) (33)	62 (GD)	YS	14	M 56 (SD 14)	29^*^	YS	NR	M 320 (SD 57)	M 940 (SD 480)	68	24	8
Schiavo *et al.* (2011, Italy) (34)	10 (GD)	Yes	36	M 49 (Rg 18–83)^*^	18^*^	No	NR	M 135 (Rg 120–150)	NR	NR	NR	50
Schiavo *et al.* (2011, Italy) (34)	17 (GD)	Yes	36	M 49 (Rg 18–83)^*^	18^*^	YS	NR	M 175 (Rg 150–200)	NR	NR	NR	41
Schiavo *et al.* (2011, Italy) (34)	92 (GD)	Yes	36	M 49 (Rg 18–83)^*^	18^*^	YS	NR	M 225 (Rg 200–250)	NR	NR	NR	13
Schiavo *et al.* (2013, Italy) (36)	75 (HTN)	Yes	30	Md 69 (Rg 31–87)	39	No	NR	300 (to nodule)	Rg 92–600	8	91	1
Schiavo *et al.* (2014, Italy) (35)	93 (TNG)	Yes	60	Md 71 (Rg 43–84)	30	No	NR	M 275 (Rg 250–300)	Md 526 (Rg 156–625)	13	69	18
Willemsen *et al.* (1993, Germany) (37)	43 (GD)	Yes	3	NR	16	YS	YS	300	Md 752 (Rg 240–3120)	63	23	14
Willemsen *et al.* (1993, Germany) (37)	43 (GD)	Yes	6	NR	16	YS	YS	300	Md 752 (Rg 240–3120)	NR	NR	7
Willemsen *et al.* (1993, Germany) (37)	43 (GD)	Yes	12	NR	16	YS	YS	300	Md 752 (Rg 240–3120)	NR	NR	0
Willemsen *et al.* (1993, Germany) (37)	43 (GD)	Yes	18	NR	16	YS	YS	300	Md 752 (Rg 240–3120)	93	7	0

If results were not reported for the different groups, for example, for different radiation absorbed dose groups or for patients grouped by disease type, the population result was presented and is indicated by asterisk.

#, number of study subjects; CI, 95% confidence interval; ATD, use of Antithyroid drugs during radioiodine administration; Eu, euthyroidism outcome at follow-up; EuG, euthryoid goiter; FU, reported follow-up time; GD, Graves' disease; HN, homogeneous uptake with no indication of GD; HTN, hyperfunctioning thyroid nodules; hyper, hyperthyroidism outcome or further radioiodine treatment at follow-up; hypo, hypothyroidism outcome at follow-up; M, mean; Md, median; NA, not applicable to study; NR, not reported in study; OP, presence of ophthalmopathy in study population; Prev RAI, previous radioactive iodine administrations; Q25, 25th quartile; Q75, 75th quartile; Rg, range; Rad Act Admin, radioactivity administered to patients; SD, standard deviation; TA, toxic adenoma; TNG, toxic nodular goiter; Yes, yes-all, that is, applicable to the full study population; YS, yes-some, that is, only applicable to a fraction of the study population.

One study (24), comprising a mixture of hyperthyroid conditions, was excluded from the quantitative synthesis due to a high risk of bias identified from the critical appraisal checklist developed by the Joanna Briggs Institute. The remaining studies were classed as low or intermediate risk of bias (Table A1 in Supplementary Data). A total of 2328 patients were reported as having Graves' disease, while 75, 173, and 57 patients had thyroid nodules, toxic nodular goiter or toxic adenoma, respectively.

Only four studies included patients with hyperfunctioning thyroid nodules or toxic nodular goiter, which was insufficient to perform a meta-analysis.

Of the studies reporting outcomes for Graves' disease, the subpopulations, as stratified by radiation absorbed dose, ranged in size from 9 to 284 patients, with a median of 42 patients. The stated aim of treatment varied between studies. In eight studies, the aim was to resolve hyperthyroidism by rendering patients either euthyroid or hypothyroid. In 4 studies, the aim was to explicitly induce euthyroidism, in 1 study, the aim was to induce hypothyroidism, and in 5 studies, the aim was not clearly reported.

A range of dosimetry methodologies (Table A3 in Supplementary Data) were employed across the studies reporting outcomes for Graves' disease, with the majority (15/18) using a variation of the method proposed by Marinelli (38), which has been adopted into EANM guidelines (3,7). Two studies (27,34) used a method based on the volume-reduction methodology proposed by Traino *et al.* (39) and one study used a fixed activity administration with post-therapy dosimetry (26). Seven studies carried out post-therapy verification, whereas 11 studies based the reported radiation absorbed dose on a pretherapy tracer study.

One study excluded patients with ophthalmopathy (31), while one study adjusted the prescribed radiation absorbed dose based on the presence of ophthalmopathy (34). Only one study reported outcomes separately for patients with ophthalmopathy (32). Less than one-third (5/18) of studies included a last follow-up of >12 months. The median last follow-up was 12 months (range 3–120 months).

For studies reporting outcomes for Graves' disease, a forest plot for the nonhyperthyroid outcome is included in the Supplementary Data (Fig. B1). The random-effects meta-analysis for this outcome resulted in an I^2^ of 91.1%, suggesting that a pooled estimate of proportion across these studies is of limited use. A strong association was found in meta-regression between the radiation absorbed dose to the thyroid and nonhyperthyroid and hypothyroid outcomes at the last reported follow-up (odds ratio [OR] = 1.11 [CI 1.08–1.14] and OR = 1.09 [CI 1.06–1.12] per 10 Gy increase in radiation absorbed dose, respectively, *R*^2^ = 55.0% and 53.7%, both *p* < 0.001).

**FIG. 2.**
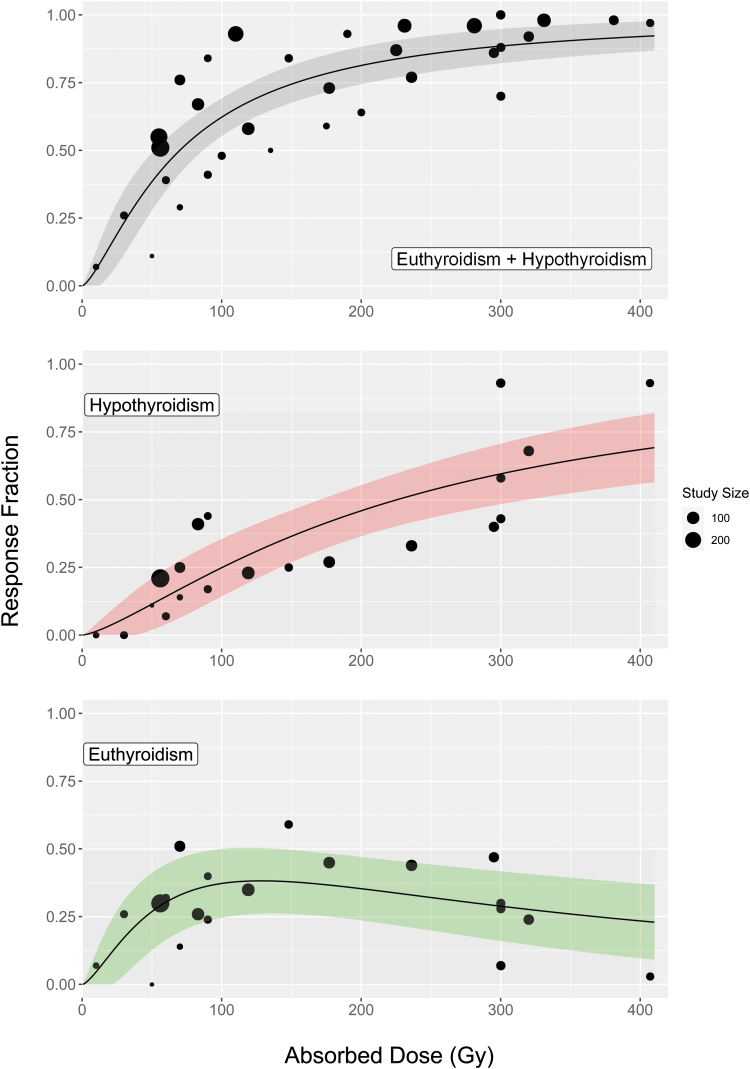
The population fraction achieving nonhyperthyroid, hypothyroid, and euthyroid outcomes as a function of radiation absorbed dose at a median follow-up of 12 months for patients with Graves' disease. The top figure represents a total of 2303 patients while the two bottom figures each represent a total of 1172 patients. The size of each point represents the number of patients in the study. The shaded regions give the 95% confidence interval. Color images are available online.

The absorbed radiation dose–response relationships for each outcome are shown in Figure 2. Given that, in the majority of studies, the administered radioactivity was calculated to deliver a prescribed radiation absorbed dose to the thyroid, these two variables are not independent (Pearson correlation coefficient *r*[15] = 0.85, *p* < 0.001). A graph of administered radioactivities against prescribed radiation absorbed doses is presented in the Supplementary Data (Fig. B2). As a result, administered radioactivity was excluded from the univariate analysis.

The proportion of patients with nonhyperthyroid and hypothyroid outcomes was seen to plateau with increasing radiation absorbed doses, with limited benefit >300 Gy (Fig. 2). An association with euthyroid outcome was found for radiation absorbed doses within the range 120–180 Gy when compared with those outside this range (*n* = 1172, OR = 2.50 [CI 1.17–5.35], *p* = 0.018). A maximum euthyroid response of 38% [CI 26–50%] was identified at a radiation absorbed dose of 128 Gy. Euthyroid, hypothyroid, and nonhyperthyroid responses at 150, 200, and 300 Gy are presented in Table 2. All ORs calculated in the sensitivity analysis (Table A2 in Supplementary Data) agreed with the results in the full analysis to within the stated CIs.

**Table 2.** Euthyroid, Hypothyroid, and Nonhyperthyroid Responses at 150, 200, and 300 Gy

Radiation absorbed dose to thyroid [Gy]	Euthyroid [%]	Hypothyroid [%]	Nonhyperthyroid [%]
150	38 [CI 26–50]	36 [CI 27–46]	74 [CI 68–81]
200	35 [CI 24–47]	46 [CI 36–55]	81 [CI 74–88]
300	29 [CI 16–42]	59 [CI 48–71]	88 [CI 82–95]

## Discussion

The findings of this systematic review and meta-analysis of 15 studies (16,20–22,25–34,37) that reported outcomes of radioiodine treatment for the subpopulation of patients with Graves' disease (*n* = 2303) indicate that there is a clear relationship between the radiation absorbed dose delivered to the thyroid and treatment outcome. This offers the potential to treat according to a desired outcome, considering potential risk factors (40,41). While EANM guidelines suggest that dosimetry-based treatment is feasible (3), other professional societies consider such an approach unviable and unproven (2,4,5).

These findings indicate that a radiation absorbed dose to the thyroid of 128 Gy achieves a euthyroid state, without the need for thyroid hormone replacement drugs, in 38% of patients and resolution of hyperthyroidism in 70% of patients at a median follow-up of 12 months. The remaining 30% of patients would require further treatment to resolve hyperthyroidism. Several studies have shown that unresolved hyperthyroidism is associated with increased risk of cardiovascular mortality (42,43). Therefore, if the clinical priority is resolution of hyperthyroidism, a higher population response rate can be achieved with a higher radiation absorbed dose. However, this will result in more patients becoming hypothyroid.

To achieve euthyroidism rates higher than 38%, personalized radiation absorbed dose prescriptions based on patient-specific factors such as the radiation absorbed dose rate (44), sex (8), thyroid volume (45), presenting triiodothyronine (8), antithyroid medication (46), and duration of the Graves' disease (47) may be required. The exact role of these factors should be further investigated.

The studies in this review show that, while administered radioactivity and radiation absorbed dose are related, different patients required different amounts of radioactivity to deliver a prescribed radiation absorbed dose to the thyroid (Fig. B2 in Supplementary Data) (16,19–24,27–29,31,33,35–37). Conversely, the administration of empirically determined standard amounts of radioactivity delivers a wide range of radiation absorbed doses to the thyroid (16,26), which results in varying response rates (Fig. 2).

Limitations of the study include the lack of data from RCTs, with only one RCT included (16). Treatment outcomes were not reported at consistent follow-up times across the studies, therefore, outcomes at last follow-up were used in our meta-analysis. The median last follow-up at 12 months may not represent the longer term effect of treatment with radioiodine. It has been shown that incidence of hypothyroidism increases with time after treatment, although this may plateau out (29). However, follow-up time was not found to be significantly associated with outcome in our meta-analysis. Further studies with long-term follow-up are required to determine how long the euthyroid state can be maintained after radioiodine treatment. Dosimetry methodologies vary between studies, which partially explains the observed variation in response rates for a given radiation absorbed dose.

Standardization of dosimetry methodology between centers, which has shown to be feasible (48), would contribute toward reducing this variation in future studies. The lack of available data for other hyperthyroid conditions limited the scope of the meta-analysis to Graves' disease. No patient-specific covariates could be extracted as they were either missing or only reported as population averages. The effect of follow-up time and patient-specific factors such as disease type, thyroid volume, or free triiodothyronine on treatment outcome should be investigated in future studies.

## Conclusions

In this study, a highly significant relationship was demonstrated between radiation absorbed dose and nonhyperthyroid, euthyroid, and hypothyroid outcomes in the treatment of Graves' disease using radioiodine. This could, therefore, serve as a basis to plan treatment, based on the required outcome. Comprehensive and standardized data collection in future studies would benefit the field. Further studies are required to determine the clinical efficacy and cost-effectiveness of dosimetry-based patient-specific treatment planning and to further investigate the potential role of patient-specific covariates that may be used for stratification.

## Supplementary Material

Supplemental data
