# Supplementary material for: A Systematic Review and Meta-Analysis of the Relationship Between the Radiation Absorbed Dose to the Thyroid and Response in Patients Treated with Radioiodine for Graves' Disease
Source: Thyroid. 2021 Dec 16;31(12):1829–38. doi: 10.1089/thy.2021.0302 (PMC8721505; doi:10.1089/thy.2021.0302)

Citation

Jan Taprogge, Glenn Flux, Lily Carnegie-Peake, Paul Gape. A systematic review and meta-analysis of the relationship between the absorbed dose delivered to the thyroid and response in patients treated with radioiodine for benign thyroid diseases. PROSPERO 2020 CRD42020175010 Available from: https://www.crd.york.ac.uk/prospero/display_record.php?ID=CRD42020175010

Review question

What is the evidence for a relationship between the absorbed radiation dose to the thyroid and the clinical outcome of patients (defined as hypothyroidism, euthyroidism or hyperthyroidism at follow-up) treated with 131I-NaI (radioiodine) for benign thyroid conditions?

Searches

PubMed, Web of Science, OVID MEDLINE and Embase. Search date of all databases: 13/02/2020

Restrictions applied to all searches:

- No language restrictions were applied for the database searches

- Any publications published before 13/02/2020 were included in the search.

Types of study to be included

No restrictions on the types of study design eligible for inclusion. Only studies will be included that report the radiation dose to the thyroid, the follow-up times at which patient outcome was assessed and which report proportion of patients at one or more follow up times which are no longer classed as being hyperthyroid.

Condition or domain being studied

Condition to be studied: Hyperthyroidism.

Hyperthyroidism is a medical condition that results in an excess amount of thyroid hormones to be produced by the thyroid gland. Iodine-131-NaI (radioiodine) has been used for the treatment of hyperthyroidism since 1941 when it was first used by Hertz and Roberts [1].

[1] Fahey, F.H., F.D. Grant, and J.H. Thrall, Saul Hertz, MD, and the birth of radionuclide therapy. EJNMMI Phys, 2017. 4(1): p. 15.

Participants/population

Inclusion:

- Adults with benign thyroid conditions (hyperthyroidism).

Exclusion:

- Subjects under 18 years of age.

Intervention(s), exposure(s)

Intervention to be reviewed: The use of Iodine-131-NaI (radioiodine) for the treatment of hyperthyroidism.

Current guidelines provided by professional societies supply conflicting principles on the optimal strategy to determine the activity of radioiodine to be used. The advocated methodologies range from employing empirical activities to complex dose calculations [2-7]. With the recently implemented European Council Directive 2013/59 [8] and the publication of ICRP 140 [9] it appears mandatory to reinvestigate the current status of evidence for performing patient tailored treatment planning based on the absorbed dose to the thyroid.

[2] Stokkel, M.P., et al. EJNMMI, 2010. 37(11): p. 2218-28.
[3] Jonklaas, J., et al. Thyroid, 2014. 24(12): p. 1670-751.
[4] Hanscheid, H., et al. EJNMMI, 2013. 40(7): p. 1126-34.
[5] Royal College of Physicians. Report of a Working Party. London: RCP, 2007.
[6] Kahaly, G.J., et al. European Thyroid Journal, 2018. 7(4): p. 167-186.
[7] Bahn Chair, R.S., et al. Thyroid, 2011. 21(6): p. 593-646.
[8] Council Directive 2013/59/Euratom. Official Journal of the European Union, 2014. 57.
[9] ICRP. ICRP Publication 140. Ann. ICRP, 2019. 48(1).

Comparator(s)/control
Not applicable.

Main outcome(s)
To assess the relationship between the absorbed dose to the thyroid of patients with hyperthyroidism and treatment outcome.

Measures of effect

Spearman's rank correlation coefficient for absorbed dose to the thyroid and treatment outcome.

Additional outcome(s)
None.

Measures of effect

Not applicable.

Data extraction (selection and coding)

Studies obtained from the initial search will be reviewed by two blinded reviewers based on title and abstract. After finishing the initial screening, results will be collated and any discrepancies between the selected studies will be resolved as a joint decision by both reviewers. Any remaining studies will then be assessed based on the full text of the publication.

Studies will be excluded at this stage if they do not report the follow-up period of patients, the radiation dose to the thyroid or the percentage of patients remaining hyperthyroid at follow-up.

For any included study, the following data will be record in an Microsoft Excel spreadsheet independently by the two blinded reviewers:
a. Main author
b. Journal/Edition/Pages
c. Year
d. Title
e. Type of study
f. Number of subjects in study group

g. Disease type
h. Discontinuation of anti-thyroid medication before treatment
i. Follow up period [in months]
j. Thyroid weight determination methodology
k. Mean Age [in years]
l. Percentage of patients being male or females
m. Radiation dose to thyroid [in Gy]
n. Details of dosimetry methodology (Number of time points, calculation methods, measurement types)
o. Percentage of patients euthyroid at follow-up
p. Percentage of patients hypothyroid at follow-up
q. Percentage of patients hyperthyroid at follow-up

Risk of bias (quality) assessment

Risk of bias will be assessed by two reviewers (JT, GF) and disagreement between the two reviewers will be resolved by consensus. The majority of studies are expected to be case series and the Critical Appraisal Checklist developed by the Joanna Briggs Institute will be used to assess the methodological quality of the included studies. Studies will be classed as include or exclude for the further data synthesis.

Strategy for data synthesis

Any studies that report follow-up period of patients, the radiation dose to the thyroid and the percentage of patients remaining hyperthyroid at follow-up will be included in the data synthesis and that have been classed as "include" using the critical appraisal checklist.

Data synthesis will be performed by a single reviewer (JT) and results will be checked for consistency by a second reviewer (GF).

A quantitative synthesis to assess the dose-response pattern(s) is performed across the selected studies. We will assess the relationship between the absorbed dose to the thyroid of patients [in Gy] and treatment outcome [Percentage of patients no longer hyperthyroid]. This analysis will be performed separately for Graves' Disease patients and any other hyperthyroid conditions (i.e. toxic adenoma, toxic multinodular goiter), if appropriate. The relationships will be assessed using appropriate statistical tests in R software for absorbed dose to the thyroid and treatment outcome.

Results will be interpreted to assess the possibility of a dose-response relationship in the treatment of these patients.

Analysis of subgroups or subsets

No sub-group analysis planned.

Contact details for further information

Jan Taprogge
jan.taprogge@nhs.net

Organisational affiliation of the review

Royal Marsden Hospital NHSFT
www.royalmarsden.nhs.uk

Review team members and their organisational affiliations

Dr Jan Taprogge. Royal Marsden Hospital NHSFT
Dr Glenn Flux. Royal Marsden Hospital NHSFT
Lily Carnegie-Peake. Royal Marsden Hospital NHSFT
Mr Paul Gape. Royal Marsden Hospital NHSFT

Type and method of review
Meta-analysis, Systematic review

Anticipated or actual start date
03 February 2020

Anticipated completion date
01 May 2021

Funding sources/sponsors
None.

Grant number(s)

State the funder, grant or award number and the date of award

Conflicts of interest
The other authors declare that they have no known conflicts of interest.
None known

Language
English

Country
England

Stage of review
Review Ongoing

Subject index terms status
Subject indexing assigned by CRD

Subject index terms
Humans; Iodine Radioisotopes; Thyroid Diseases

Date of registration in PROSPERO
21 May 2020

Date of first submission
31 March 2020

Details of any existing review of the same topic by the same authors

Stage of review at time of this submission

Stage	Started	Completed
Preliminary searches	Yes	Yes
Piloting of the study selection process	Yes	Yes
Formal screening of search results against eligibility criteria	Yes	Yes
Data extraction	Yes	No
Risk of bias (quality) assessment	Yes	No
Data analysis	No	No

Revision note

Updated to change "prescribed radiation dose" to "radiation dose" as the dose to the thyroid was not prescribed in the studies. Updated to change any reference to "at end of follow-up" to "at follow-up" as some studies report multiple follow up times.Updated to change the statistical tests performed prior to start of data analysis.

The record owner confirms that the information they have supplied for this submission is accurate and complete and they understand that deliberate provision of inaccurate information or omission of data may be construed as scientific misconduct.
The record owner confirms that they will update the status of the review when it is completed and will add publication details in due course.

Versions

21 May 2020
27 November 2020
23 March 2021

Joanna Briggs Questionnaire (including additional question 11)

1· Were there clear criteria for inclusion in the case series?

2· Was the condition measured in a standard, reliable way for all participants included in the case series?

3· Were valid methods used for identification of the condition for all participants included in the case series?

4· Did the case series have consecutive inclusion of participants?

5· Did the case series have complete inclusion of participants?

6· Was there clear reporting of the demographics of the participants in the study?

7· Was there clear reporting of clinical information of the participants?

8· Were the outcomes or follow-up results of cases clearly reported?

9· Was there clear reporting of the presenting site(s)/clinic(s) demographic information?

10· Was statistical analysis appropriate?

11· Are treatment methodologies in all sub-groups comparable and match other studies?

Table A1: Risk of Bias Assessment

	**Question**											**Risk of bias**
	**1**	**2**	**3**	**4**	**5**	**6**	**7**	**8**	**9**	**10**	**11**	
Amato et al (2016)	Y	Y	Y	Y	Y	Y	Y	Y	NR	NR	Y	Low
Bajnok et al (1999)	Y	Y	Y	Y	Y	Y	Y	Y	Y	Y	Y	Low
Berg et al (1996)	Y	Y	NR	Y	N	Y	Y	Y	Y	Y	Y	Low
Blahd et al (1972)	Y	Y	Y	NR	NR	Y	N	Y	Y	NR	Y	Intermediate
Bockisch et al (1993)	Y	Y	Y	N	N	N	Y	Y	N	NR	Y	Intermediate
Camps et al (1996)	Y	NR	Y	Y	N	Y	Y	N	N	NR	N	High
Catargi et al (1999)	Y	Y	Y	NR	N	Y	Y	Y	N	Y	Y	Low
Flower et al (1994)	Y	Y	Y	Y	N	N	Y	Y	N	NR	N	Intermediate
Grosso et al (2005)	Y	Y	Y	NR	NR	Y	Y	Y	N	Y	Y	Low
Howarth et al (2001)	Y	Y	Y	N	NR	Y	Y	Y	Y	Y	Y	Low
Hyer et al (2018)	Y	Y	Y	Y	N	Y	Y	Y	NR	Y	Y	Low
Kobe et al (2008)	Y	Y	Y	Y	N	Y	Y	Y	Y	Y	Y	Low
Orsini et al (2012)	Y	Y	Y	Y	Y	N	Y	Y	Y	Y	Y	Low
Oszukowska et al (2010)	Y	Y	Y	NR	NR	Y	Y	Y	Y	Y	Y	Low
Peters et al (1995)	Y	Y	Y	N	N	Y	Y	Y	Y	Y	Y	Low
Reinhardt et al (2002)	Y	Y	Y	Y	Y	Y	Y	Y	Y	Y	Y	Low
Schiavo et al (2011)	Y	Y	Y	Y	NR	N	Y	Y	N	Y	Y	Intermediate
Schiavo et al (2013)	Y	Y	Y	Y	Y	Y	Y	Y	Y	Y	Y	Low
Schiavo et al (2014)	Y	Y	Y	Y	Y	Y	Y	Y	Y	Y	Y	Low
Willemsen et al (1993)	Y	Y	Y	NR	NR	N	Y	Y	Y	Y	Y	Intermediate

Table A2: Results of the sensitivity analysis to identify whether results remained significant when only including studies classed as having low risk of bias. NA = not applicable.

	**Studies Included**	**Covariates**	**Regression Coefficient**	**95% CI (Lower)**	**95% CI (Upper)**	**OR (per 10 Gy)**	**95% CI (Lower)**	**95% CI (Upper)**	**p value**
Non-Hyperthyroid	Low+Intermediate Bias Risk (k=32)	Intercept	-0·654	-1·207	-0·101	NA	NA	NA	0·0205
		Absorbed Dose (Gy)	0·011	0·008	0·013	1·11	1·08	1·14	<·0001
	Low Bias Risk (k=23)	Intercept	-0·110	-0·848	0·627	NA	NA	NA	0·7692
		Absorbed Dose (Gy)	0·008	0·005	0·012	1·09	1·05	1·13	<·0001
Hypothyroid	Low+Intermediate Bias Risk (k=20)	Intercept	-2·060	-2·653	-1·467	NA	NA	NA	<·0001
		Absorbed Dose (Gy)	0·008	0·006	0·011	1·09	1·06	1·12	<·0001
	Low Bias Risk (k=15)	Intercept	-1·714	-2·275	-1·153	NA	NA	NA	<·0001
		Absorbed Dose (Gy)	0·006	0·004	0·009	1·07	1·04	1·09	<·0001
Euthyroid	Low+Intermediate Bias Risk (k=20)	Intercept	-0·865	-1·134	-0·596	NA	NA	NA	<·0001
		Absorbed Dose 120 – 180 Gy	0·916	0·161	1·294	2·50	0·16	1·68	0·0182
	Low Bias Risk (k=15)	Intercept	-0·756	-1·043	-0·468	NA	NA	NA	<·0001
		Absorbed Dose 120 – 180 Gy	0·527	0·013	1·041	1·69	1·01	2·83	0·0447

Table A3: Summary of dosimetry methodologies used in the papers assessed in the present systematic review. Dosimetry formulas used refer to Marinelli et al (Am J Roentgenol Radium Ther. 1948;59(2):260-81) or variations of the original formula proposed by Marinelli et al and Traino et al (Nucl Med Commun. 2006;27(5):439-446).

	Formula	Pre-therapy tracer study	Post-therapy verification	Uptake measurement equipment used	Time points (Tracer study) [hours]	Time points (Verification) [hours]	Volume determination methodology
Amato et al (2016)	Marinelli	Yes (^{131}I-NaI)	No	Scintillation probe	3, 6, 24, 48, 72 or 96, and 168	N/A	US
Bajnok et al (1999)	Marinelli	Yes (^{131}I-NaI)	No	Not stated	24 and 120 to 168	N/A	Scintigraphy (^{99m}Tc-pertechnetate)
Berg et al (1996)	Modified Marinelli	Yes (^{131}I-NaI)	No	Not stated	24, 48 and 96 or 144	N/A	Scintigraphy (^{99m}Tc-pertechnetate)
Blahd et al (1972)	Modified Marinelli	Yes (^{131}I-NaI)	No	Not stated	Not stated	N/A	Scintigraphy (Isotope not stated)
Bockisch et al (1993)	Modified Marinelli	Yes (^{131}I-NaI)	Yes	Scintillation probe	8, 24, 32, 48, 72, 96 and 192	8, 24, 32, 48, 72, 96 and 192	US combined with scintigraphy
Camps et al (1996)	Modified Marinelli	Yes (^{123}I-NaI)	No	Not stated	24 and 120	N/A	Scintigraphy (^{123}I-NaI gamma camera imaging)
Catargi et al (1999)	Modified Marinelli	Yes (^{131}I-NaI)	Yes	Scintillation probe	3, 6, 24 and 48	24, 48, 72, 96, 120	US
Flower et al (1994)	Fixed activity with post-therapy dosimetry	No	Yes	Scintillation probe	N/A	24 and 120	^{124}I-NaI PET
Grosso et al (2005)	Traino	Yes (^{131}I-NaI)	No	Scintillation probe	4 and 24	N/A	US
Howarth et al (2001)	Modified Marinelli	Yes (^{131}I-NaI)	No	Scintillation probe	24 and 168	N/A	US
Hyer et al (2018)	Modified Marinelli	Yes (^{131}I-NaI)	Yes	Scintillation probe	24, 48 and 144	24 (Half-life from tracer study)	US
Kobe et al (2008)	Marinelli	Yes (^{131}I-NaI)	Yes	Scintillation probe	6, 24 and 120	Not stated	US
Orsini et al (2012)	Marinelli	Yes (^{131}I-NaI)	No	Scintillation probe	4, 24, 48 and 96	N/A	US
Oszukowska et al (2010)	Marinelli	Yes (^{131}I-NaI)	No	Scintillation probe	24, 48 and 72	N/A	US
Peters et al (1995)	Marinelli	Yes (^{131}I-NaI)	Yes	Not stated	Not stated	Not stated	US
Reinhardt et al (2002)	Marinelli	Yes (^{131}I-NaI)	Yes	Scintillation probe	Not stated	Not stated	US
Schiavo et al (2011)	Traino	Yes (^{131}I-NaI)	No	Scintillation probe	4, 24 and 96-120	N/A	US
Schiavo et al (2013)	Traino	Yes (^{131}I-NaI)	No	Scintillation probe	4, 24 and 96-120	N/A	US
Schiavo et al (2014)	Traino	Yes (^{131}I-NaI)	No	Scintillation probe	4, 24 and 96-120	N/A	US
Willemsen et al (1993)	Marinelli	Yes (^{131}I-NaI)	No	Not stated	6, 24, 48 and 72	N/A	US

Figure B1: Forest plot of pooled proportion of patients reaching non-hyperthyroid outcome following radioiodine treatment. (* = outcomes reported separately for patients with Graves' ophthalmopathy)

Study	Events	Total	Proportion	95%-CI	Weight
Bajnok (70 Gy)	52	68	0.76	[0.65; 0.86]	3.6%
Bajnok (90 Gy)	21	25	0.84	[0.64; 0.95]	3.0%
Berg (110 Gy)	178	191	0.93	[0.89; 0.96]	3.6%
Blahd (55 Gy)	132	241	0.55	[0.48; 0.61]	3.8%
Catargi (83 Gy)	67	100	0.67	[0.57; 0.76]	3.7%
Flower (10 Gy)	1	15	0.07	[0.00; 0.32]	1.9%
Flower (30 Gy)	7	27	0.26	[0.11; 0.46]	3.3%
Flower (50 Gy)	1	9	0.11	[0.00; 0.48]	1.9%
Flower (70 Gy)	4	14	0.29	[0.08; 0.58]	2.9%
Grosso (148 Gy)	27	32	0.84	[0.67; 0.95]	3.2%
Grosso (295 Gy)	50	58	0.86	[0.75; 0.94]	3.4%
Howarth (60 Gy)	11	28	0.39	[0.22; 0.59]	3.4%
Howarth (90 Gy)	12	29	0.41	[0.24; 0.61]	3.4%
Hyer (60 Gy)	146	284	0.51	[0.45; 0.57]	3.9%
Kobe (190 Gy)	28	30	0.93	[0.78; 0.99]	2.6%
Kobe (231 Gy)	132	137	0.96	[0.92; 0.99]	3.3%
Kobe (281 Gy)	173	181	0.96	[0.91; 0.98]	3.5%
Kobe (331 Gy)	125	128	0.98	[0.93; 1.00]	2.9%
Kobe (381 Gy)	49	50	0.98	[0.89; 1.00]	2.0%
Orsini (100 Gy)	14	29	0.48	[0.29; 0.67]	3.4%
Orsini (200 Gy)	16	25	0.64	[0.43; 0.82]	3.3%
Orsini (407 Gy)	28	29	0.97	[0.82; 1.00]	2.0%
Oszukowsa (300 Gy)	35	40	0.88	[0.73; 0.96]	3.2%
Oszukowsa (300 Gy)*	28	40	0.70	[0.53; 0.83]	3.5%
Peters (119 Gy)	62	107	0.58	[0.48; 0.67]	3.8%
Reinhardt (177 Gy)	61	84	0.73	[0.62; 0.82]	3.7%
Reinhardt (236 Gy)	60	78	0.77	[0.66; 0.86]	3.6%
Reinhardt (320 Gy)	57	62	0.92	[0.82; 0.97]	3.2%
Schiavo (135 Gy)	5	10	0.50	[0.19; 0.81]	2.8%
Schiavo (175 Gy)	10	17	0.59	[0.33; 0.82]	3.2%
Schiavo (225 Gy)	80	92	0.87	[0.78; 0.93]	3.6%
Willensen (300 Gy)	43	43	1.00	[0.92; 1.00]	1.3%
**Random effects model**		**2303**	**0.75**	**[0.67; 0.82]**	**100.0%**

Heterogeneity: $I^2 = 91\%$, $\tau^2 = 1.0362$, $p < 0.01$

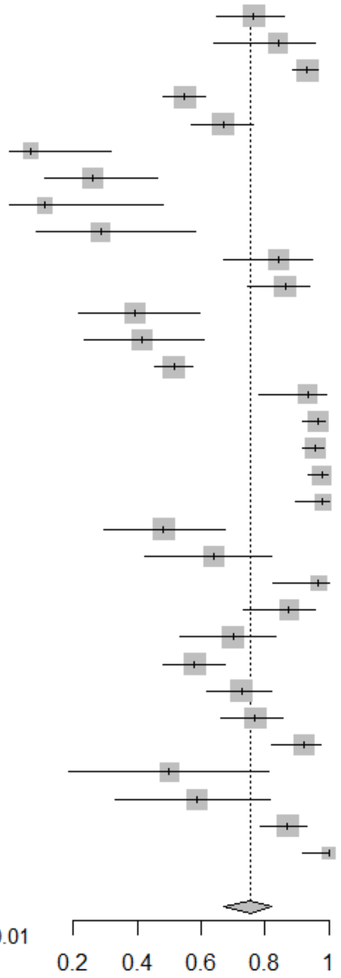

Figure B2: Relationship between administered activity and radiation absorbed dose for studies using a prescribed radiation absorbed dose methodology. Error bars represent standard deviation or 95% confidence interval, which was not consistently reported between studies. Data points without error bars are studies where no standard deviation or confidence interval was recorded for the administered activities or radiation absorbed dose.

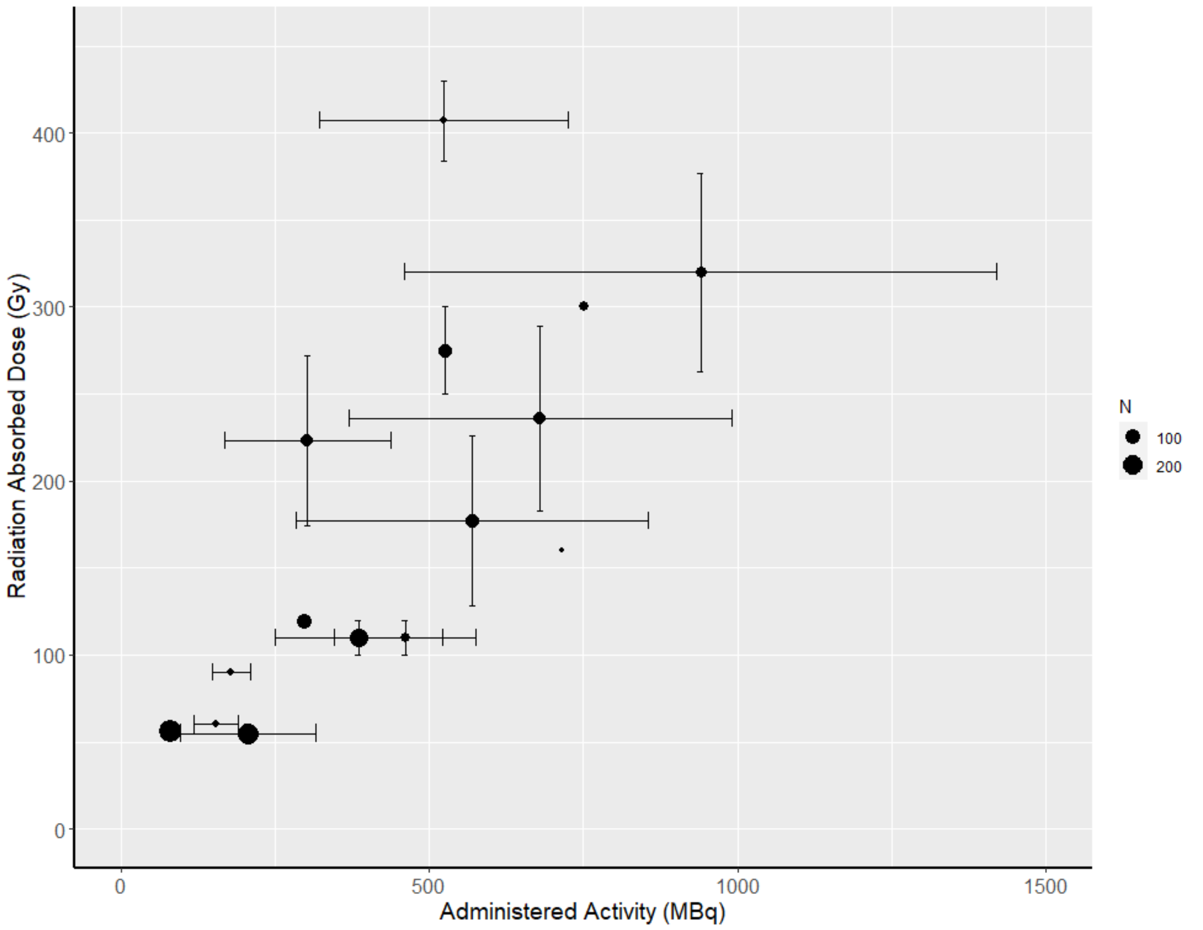

Supplement: Supplemental data [file Supp_Data.pdf]
